# Prognostic value and immune cell infiltration of hypoxic phenotype‐related gene signatures in glioblastoma microenvironment

**DOI:** 10.1111/jcmm.15939

**Published:** 2020-10-03

**Authors:** Kai Xiao, Jun Tan, Jian Yuan, Gang Peng, Wenyong Long, Jun Su, Yao Xiao, Qun Xiao, Changwu Wu, Chaoying Qin, Lili Hu, Kaili Liu, Shunlian Liu, Hao Zhou, Yichong Ning, Xiaofeng Ding, Qing Liu

**Affiliations:** ^1^ Department of Neurosurgery Xiangya Hospital of Central South University Changsha China; ^2^ Institute of Skull Base Surgery and Neuro‐oncology at Hunan Changsha China; ^3^ Institute of Anatomy University of Leipzig Leipzig Germany; ^4^ Medical College of Hunan Normal University Changsha China; ^5^ State Key Laboratory of Developmental Biology of Freshwater Fish College of Life Science Hunan Normal University Changsha China

**Keywords:** candidate targets, glioma stemness, hypoxic phenotype, immune cell infiltration, tumour microenvironment

## Abstract

Glioblastoma (GBM) is a malignant intracranial tumour with the highest proportion and lethality. It is characterized by invasiveness and heterogeneity. However, the currently available therapies are not curative. As an essential environmental cue that maintains glioma stem cells, hypoxia is considered the cause of tumour resistance to chemotherapy and radiation. Growing evidence shows that immunotherapy focusing on the tumour microenvironment is an effective treatment for GBM; however, the current clinicopathological features cannot predict the response to immunotherapy and provide accurate guidance for immunotherapy. Based on the ESTIMATE algorithm, GBM cases of The Cancer Genome Atlas (TCGA) data set were classified into high‐ and low‐immune/stromal score groups, and a four‐gene tumour environment‐related model was constructed. This model exhibited good efficiency at forecasting short‐ and long‐term prognosis and could also act as an independent prognostic biomarker. Additionally, this model and four of its genes (*CLECL5A, SERPING1, CHI3L1* and *C1R*) were found to be associated with immune cell infiltration, and further study demonstrated that these four genes might drive the hypoxic phenotype of perinecrotic GBM, which affects hypoxia‐induced glioma stemness. Therefore, these might be important candidates for immunotherapy of GBM and deserve further exploration.

## 1 INTRODUCTION

Glioblastoma (GBM) is a malignant intracranial tumour with the largest per cent of occurrence and the highest lethality; in addition, complete neurosurgical resection is not possible because of diffuse infiltration of the adjacent brain parenchyma.^1^ The standard care of GBM includes neurosurgical resection, radiotherapy and chemotherapy. Despite these additional interventions, the average median survival time is only approximately 15 months, whereas the two‐ and five‐year survival rates are approximately 26.5% and 9.7%, respectively.^2^ Immunotherapeutic strategies investigated by different approaches have shown promising results.^3^ For these therapies, it is necessary to explore the molecular signatures and candidate targets in GBM patients that affect the prognosis and immune response. The tumour microenvironment (TME), where tumour cells grow and develop, includes a series of non‐neoplastic cells and molecules, containing infiltrating and resident immune cells, vascular cells and other glial cells, with numerous cytokines and chemokines.^1^, ^4^ The dynamic changes in the TME reflect the evolutionary nature of the tumour, which involves the tumour immune escape, tumour growth and metastasis. For example, additional gene mutations induced by the low expression of DNA mismatch repair genes in a low oxygen environment could promote a more aggressive tumour phenotype.^5^ Tumour progression and therapeutic responses are significantly correlated with TME heterogeneity, thereby dictating the success of immunotherapeutic programmes.^6^ Thus, a deeper understanding of the TME and the mechanisms involved is crucial.

Innovative computational methods and genomics have given us a preliminary understanding of TME. For example, CIBERSORTx, Tumor IMmune Estimation Resource (TIMER) and Estimation of STromal and Immune cells in MAlignant Tumor tissues using Expression data (ESTIMATE) are innovative algorithms used to calculate the infiltration of tumour‐related normal cells in tumour tissues according to genomics.^7^, ^8^, ^9^ Depending on specific molecular biomarkers expressed in immune and stromal cells, the ESTIMATE algorithm computes the immune/stromal/ESTIMATE scores to reflect the TME. Researchers have successfully applied this algorithm to explore the TME of various cancers, including clear cell renal cell carcinoma, non–small cell lung cancer, acute myeloid leukaemia and colon cancer.^10^, ^11^, ^12^, ^13^ However, this algorithm is still not extensively applied to explore the GBM microenvironment in the literature.

In this study, we analysed the potential TME‐related prognostic signature for GBM patients by integrating of TCGA data and ESTIMATE algorithm, identifying differentially expressed genes based on their immune and stromal scores. The common genes were screened out and followed with a robust likelihood‐based survival model to identify prognostic genes associated with the GBM TME. Furthermore, using the LASSO method, we constructed an independent prognostic model containing four genes aberrantly expressed in GBM; these genes and the model were significantly associated with the immune infiltration levels and the hypoxic phenotype in GBM patients. The findings of this study will help elucidate the TME effect on GBM and provide alternative targets for immunotherapy.

## 2 MATERIALS AND METHODS

### 2.1 Database

The genomic expression and clinical data of GBM patients in TCGA and Gravendeel databases were retrieved from GlioVis (http://gliovis.bioinfo.cnio.es/), which is a user‐friendly website for data visualization and analysis of glioma data sets. The CGGA RNA sequencing data were downloaded from CGGA data portal (http://www.cgga.org.cn/). The stromal and immune scores of TCGA GBM data set originated from the ESTIMATE algorithm, which was downloaded from the ESTIMATE website (https://bioinformatics.mdanderson.org/estimate/), which provides information regarding tumour samples based on expression data, such as scores of tumour purity, the level of stromal cells present and the infiltration level of immune cells in tumour tissues. The file reflecting the estimation of immune cells of the TCGA GBM data set was obtained from TIMER (https://cistrome.shinyapps.io/timer/), a website offering an in‐depth analysis of infiltration of immune cells in tumours. The microarray data of 416 GBM patients with clinicopathological characteristics, stromal/immune scores and estimation of immune cells from the TCGA, normalized by the ‘affy’ package, were chosen as the training set to construct the prognostic model. The validation set included data of 159 GBM cases downloaded from Gravendeel. The clinical information for the training and validation sets is shown in Table 1. A single‐cell RNA‐seq data set comprising 3,589 cells collected from both the tumour core and the peritumoral brain was obtained from a user‐friendly website (http://www.gbmseq.org/).^14^ The single‐cell RNA‐seq data set was used to explore the expression of identified genes within the model in different cell clusters. Our workflow for this study is shown in Figure S1.

**Table 1:** Clinical parameters of patients in the training set and validation set

Variables	Training set (n = 416)	Validation set (n = 159)	*P‐*value
Age (years)			
Mean ± SD	58.1 ± 14.3	54.1 ± 14.0	0.998
Median	59.4	55.4	
Age group (median)			
Younger	206	79	1
Old	205	80	
NA (not available)	5	/	
Gender			
Female	164	51	0.09
Male	245	108	
NA	7	/	
Vital status			
Alive	60	11	0.019
Dead	353	148	
NA	3	/	
CIMP status			
G‐CIMP	33	23	0.027
Non–G‐CIMP	383	136	
MGMT status			
Methylated	134	/	
Unmethylated	143	/	
NA	139	/	
Subtype			
Classical + Mesenchymal	249	102	0.39
Neural + pro‐neural	167	57	
Classical	128	72	
Mesenchymal	121	30	
Neural	65	19	
Pro‐neural	102	38	

### 2.2 Estimating infiltration of 22 types of immune cells

CIBERSORTx is an online analytical tool based on gene expression profiles and signature matrix files that allow us to determine cell type abundance and expression from bulk tissue.^15^ The TCGA expression profile was uploaded to the online tool, and a reference LM22 signature matrix with 100 permutations was used for the tool.

### 2.3 Distinguishing and elucidating differentially expressed TME‐related genes

The median values of immune and stromal scores were selected as the cut‐off to divide the high‐ and low‐score groups. Differentially expressed genes were screened out between the high‐ and low‐score groups using the limma package.^16^ The absolute value of logFC > 1 and adjusted *P*‐value < 0.05 were fixed as the thresholds to identify differentially expressed TME‐related genes.

Functional enrichment analysis, including biological processes (BP), molecular functions (MF) and cellular components (CC), and the Kyoto Encyclopedia of Genes and Genomes (KEGG) pathway analysis were conducted via the clusterProfiler package in R language for differentially expressed TME‐related genes.^17^ Benjamini‐Hochberg (BH)–adjusted *P*‐values < 0.05 were considered statistically significant.

### 2.4 Establishing and confirming the prognostic model and building the co‐expression network of the model

Using the rbsurv, glmnet and survival packages, the robust likelihood‐based survival model and the least absolute shrinkage and selection operator regression (LASSO) analyses were conducted in the TCGA training set to construct the TME‐related model. Consequently, four prognostic TME‐related genes and their regression coefficients were obtained. Univariate and multivariate Cox hazard regression analysis showed that this gene signature model was an independent prognostic biomarker, verified using the Gravendeel validation set.

Weighted gene correlation network analysis (WGCNA), an innovative method used to describe the correlation patterns among genes across microarray samples, was conducted for the training set to build the co‐expression network of the gene signature model.^18^ The variance of each gene was calculated and sorted by number. The top 50% genes ranked based on variance were chosen for WGCNA. It means that 416 samples and 6,350 genes were included in the WGCNA. First, outliers were screened out, and a co‐expression similarity matrix was established according to the absolute value of the correlation between the expression levels of transcriptome data. Second, an adjacency matrix converted from the abovementioned matrix by choosing 5 as a soft threshold, applying the topological overlap measure, allowed co‐expression gene modules to be classified. Gene significance (GS) used to distinguish the importance of each module was computed to assess the relationship between genes and immune/stromal scores. Defined as the mean of GS within modules, module significance (MS) was estimated to measure the relationship between modules and the immune/stromal scores. Lastly, the immune/stromal score‐related gene modules containing the four genes were screened out. After filtering the genes that interplayed with the four genes, the co‐expression network of this signature was built using Cytoscape.^19^

### 2.5 Immunohistochemistry

These experiments were approved by the Human Ethics Committee of Xiangya Hospital, and informed consent was obtained from all patients. Based on polyformalin‐fixed and paraffin‐embedded tissues obtained from GBM patients, immunohistochemistry analysis was conducted as previously described.^20^ Tissue sections were incubated with indicated primary antibodies against C1R(17346‐1‐AP) and CHI3L1(12036‐1‐AP).

### 2.6 Statistical analysis

Statistical analyses were performed with R software. Using the log‐rank test, the relationship between the critical factors (gene expression level and immune/stromal scores) and patients’ overall survival was analysed based on the survival and survminer R package, with *P < *.05 regarded as statistically significant. To assess the predictive efficiency of the signature, the time‐dependent receiver operating characteristic curves were depicted.

### 2.7 Clustering single cells and identifying the role of the signature in cell clusters

Dimensionality reduction and cell clustering for the single‐cell RNA‐seq data were performed based on ‘tsne’ package in R. As described by a previous study,^14^ the cell‐to‐cell distance matrix of all cells was established based on the top 500 over‐dispersed genes. The matrix was reduced to two dimensions with tSNE. Singe cells were coloured on a dimensional reduction plot according to the genes within the model.

## 3 RESULTS

### 3.1 Immune/stromal scores remarkably correlated with molecular subtypes and prognosis of GBM

**Figure 1:**
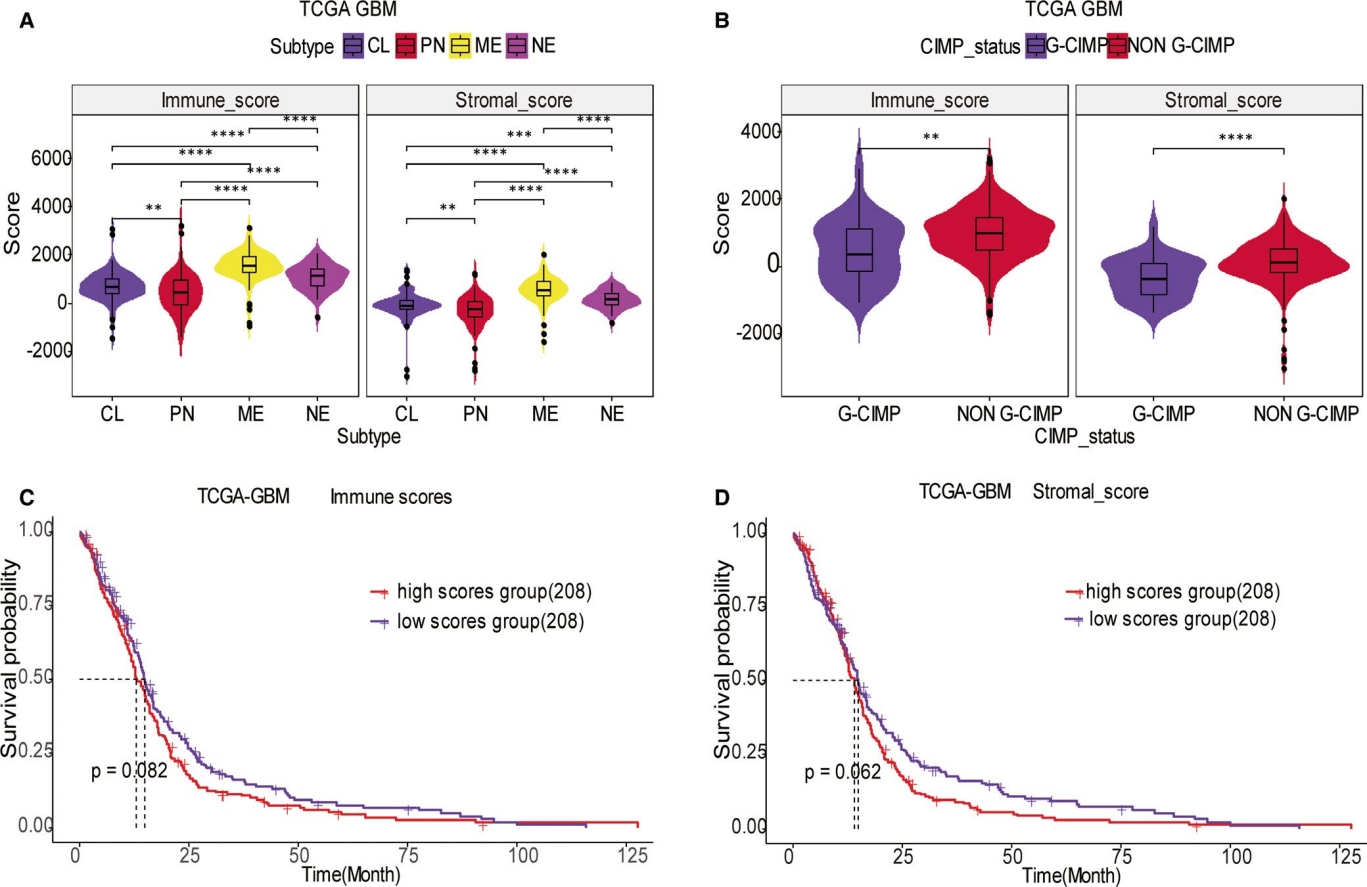
Immune score and stromal score are associated with GBM subtypes and the overall survival. (A) Distribution of immune/stromal score of TCGA GBM transcriptome subtypes. The violin plot shows that both immune score and stromal score are significantly correlated with transcriptome subtypes of GBM (n = 416, *: *P* < .05, **: *P* < .01, ***: *P* < .001). (B) Distribution of immune/stromal score of TCGA GBM non–G‐CIMP subtype and G‐CIMP subtype. The violin plot shows that both immune score and stromal score are significantly correlated with CIMP subtypes of GBM. (C) TCGA GBM cases were divided into two groups based on their immune score; median overall survival of cases with the elevated immune score is shorter than that of cases with the lower immune scores, although it was not statistically significant (*P* = .082). (D) Similarly, TCGA GBM cases were divided into two groups based on their stromal score; the median overall survival of cases with the elevated stromal score is shorter than that of the cases with the lower stromal scores, although it was not statistically significant (*P* = .062)

Of the 416 GBM cases retrieved from GlioVis transcriptome, subtyping identified 128 classical, 121 mesenchymal, 65 neural and 102 pro‐neural subtype cases. Regarding the status of CIMP, 33 patients were G‐CIMP and 383 were non–G‐CIMP. The ESTIMATE algorithm revealed the immune (−1448 to 3210.47) and stromal scores (−3055.72 to 2016.62) of the 416 GBM patients. To elucidate the relationship between the molecular expression patterns and immune/stromal scores, both scores of the four transcriptome subtypes (mesenchymal, classical, neural and preneural) and CIMP status (G‐CIMP and non–G‐CIMP) of GBMs were compared. There was a remarkable association between the immune/stromal scores and the four transcriptome subtypes. The immune/stromal scores of the mesenchymal subtype were the highest, whereas the scores of pro‐neural subtype were the lowest (Figure 1A). Immune/stromal scores in the non–G‐CIMP cluster were remarkably higher than those in the G‐CIMP cluster (Figure 1B). To study the potential relationship between immune/stromal scores and survival time of GBM patients, the median of immune and stromal scores was set as the cut‐off, and we dichotomized 416 GBM patients into low‐ and high‐score groups. The survival rate between the two clusters was compared by the Kaplan‐Meier survival analysis, separately. The outcome of survival analysis indicated that the median overall survival of cases with elevated immune/stromal scores exhibited unsatisfactory results than the cases with low‐immune/stromal scores, although the result was not remarkable (Figure 1C, *P*= .082; Figure 1D, *P* = .062). This verified that immune/stromal scores were significantly associated with the GBM subtypes and higher scores were related to shorter survival times in GBM patients. In accordance with the results from a previous study,^21^ we also found that transcriptional subtypes of IDH wild‐type tumours, except the neural subtype which was identified as a sampling artefact, differentially activated the immune microenvironment. Mesenchymal GBM exhibits a higher fraction of M2 macrophages and neutrophils and lower proportion of activated nature killer cell compared with classical and pro‐neural GBM. Meanwhile, the fraction of the resting memory CD4^+^ T cell was reduced in the pro‐neural GBM (Figure S2A).

### 3.2 Differentially expressed genes related to the GBM TME and their functional annotation

**Figure 2:**
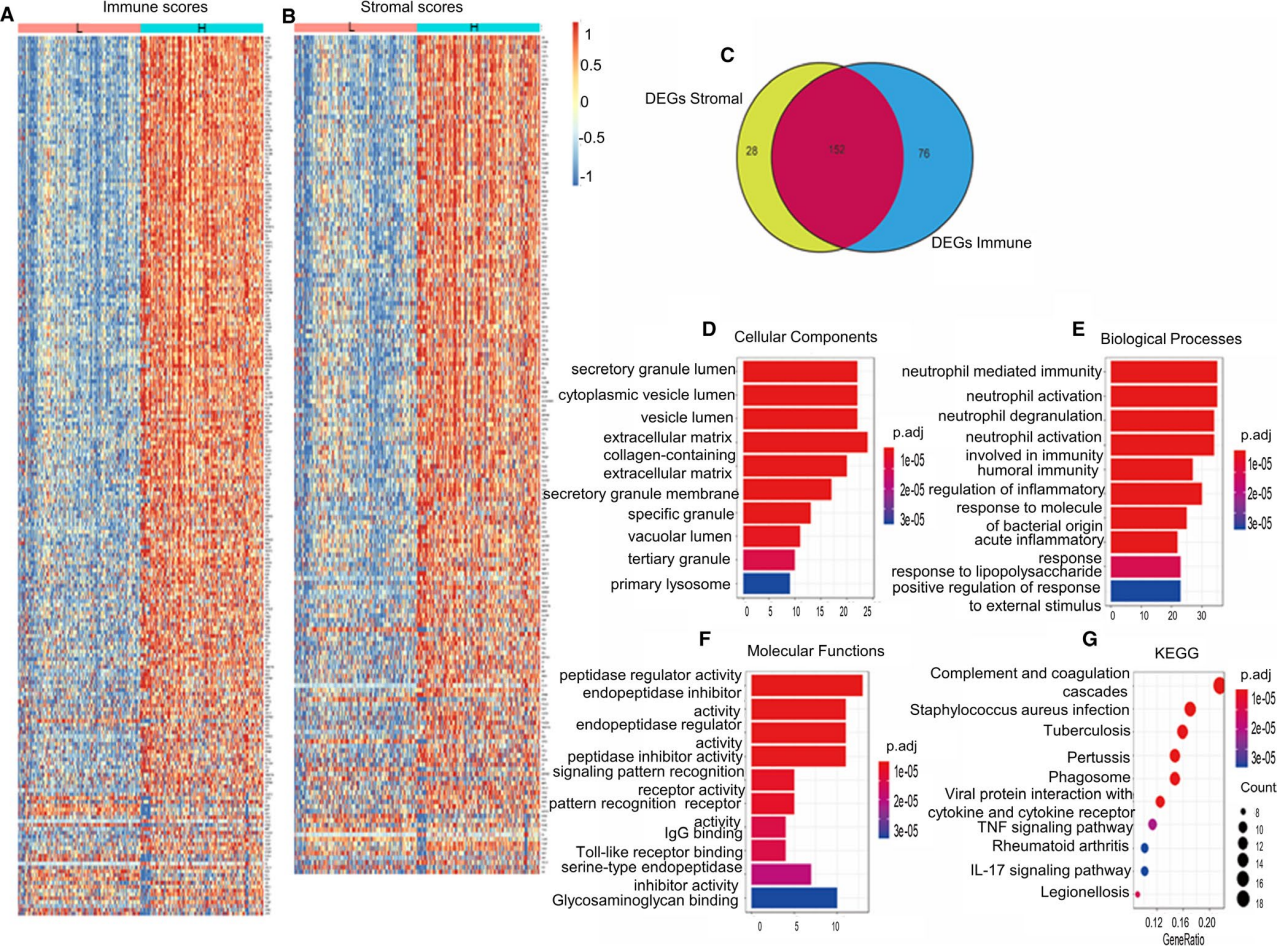
Heat map of differentially expressed genes in the high‐ and low‐immune/stromal score groups and the most significantly enriched GO annotations and KEGG pathways. The length of the bars and the size of the dots represent the numbers of genes, and the colour of the bars/dots corresponds to the p‐value according to legend. (A) Immune score (high score, right; low score, left; |log FC|> 1, *P* < .05). (B) Stromal scores (high score, right; low score, left; |log FC|> 1, *P* < .05). (C) Common differentially expressed genes detected for immune and stromal score. (D) Top 10 significantly enriched cellular components. (E) Top 10 significantly enriched biological process. (F) Top 10 significantly enriched molecular functions. (G) Top 10 significantly enriched KEGG pathways

Comparing the transcriptome data from 416 GBM patients after their separation into high‐ and low‐score groups, we attempted to uncover the correlation of gene expression profiles with immune and stromal scores. The differential gene expression profiles of patients with high‐immune/stromal scores and those with low‐immune/stromal scores can be observed in the generated heat maps (Figure 2A,B). For comparison of immune and stromal score groups, the gene expression profile showed that 228 and 180 differentially expressed genes were filtered out with the threshold adjusted *P*‐value < 0.05 and |log fold change|＞1. Moreover, Venn diagrams show 152 genes, differentially expressed both in the stromal and immune score groups (Figure 2C). Thus, we focused on these 152 genes, named differentially expressed genes related to tumour environment (DEGRTME), for the subsequent analysis. Functional enrichment analysis was conducted to clarify the mechanism underlying the functions of these 152 genes. The top 10 functional annotations of Gene Ontology (GO) terms and the KEGG terms are listed in Figure 2D‐G. The results revealed that DEGRTME were strongly associated with immune‐related terms/pathways, such as extracellular matrix, neutrophil‐mediated immunity, humoural immune response, regulation of inflammatory response, acute inflammatory response, IgG binding, signalling pattern recognition receptor activity, and complement and coagulation cascades.

### 3.3 Establishing and confirming the TME‐related model for GBMs and building the co‐expression network

**Figure 3:**
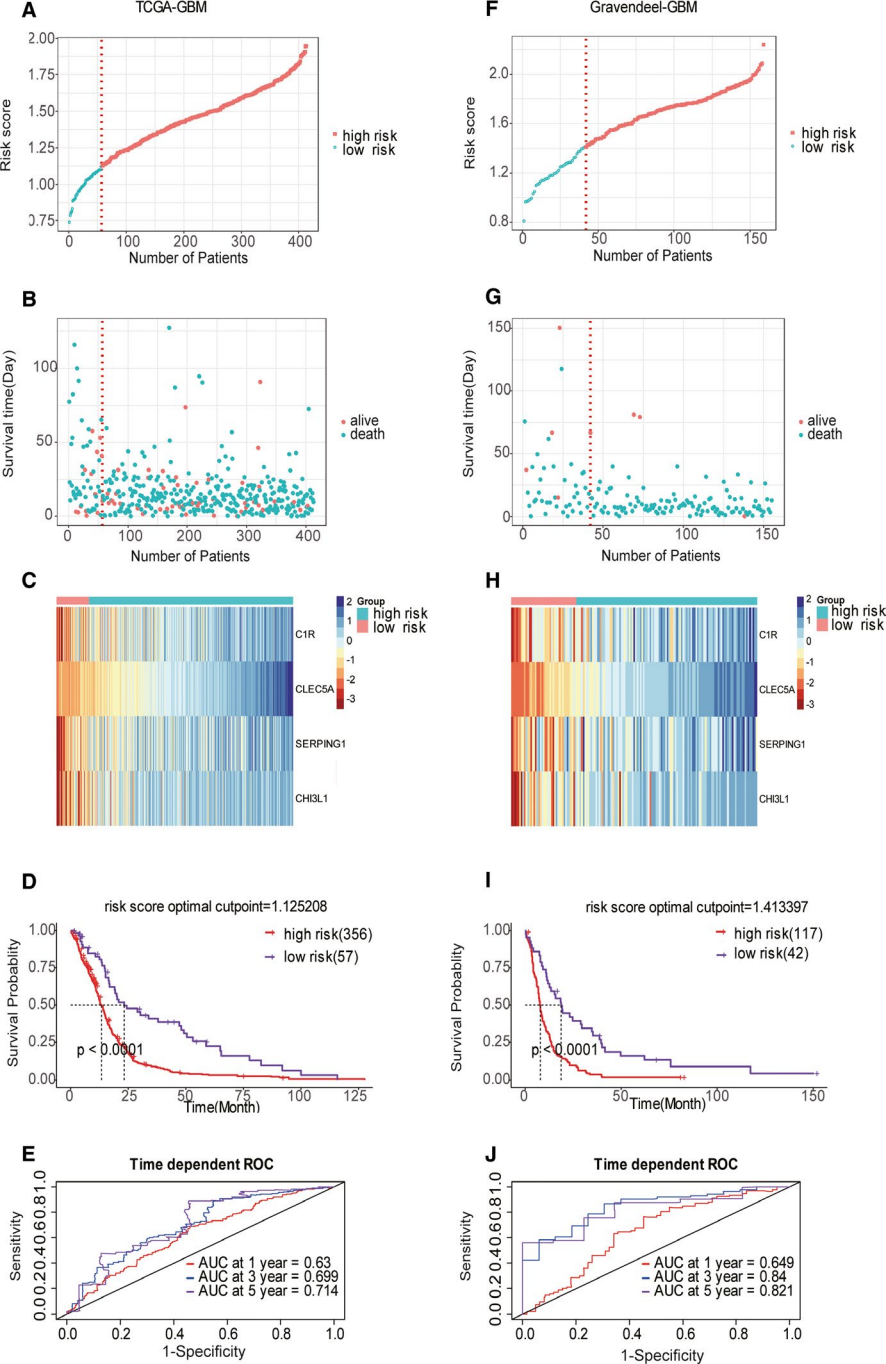
Construction and validation of four‐gene TME‐related prognostic model. (A) The four‐gene signature risk score distribution in the TCGA GBM data set. (B) Scatter plot of patient survival status ordered by risk score in the TCGA GBM data set. (C) The heat map of the four‐gene expression profiles in the TCGA GBM data set after standardization and centralization. (D) Kaplan‐Meier curve for the overall survival in the TCGA GBM cohort stratified by the four‐gene model into the high‐ and low‐risk group based on the optimal cut‐off point of the risk score. (E) Time‐dependent ROC curves indicated good performance of our prognostic model in the TCGA GBM cohort. (F‐J) The above‐mentioned results can be noted in the Gravendeel validation data set

**Figure 4:**
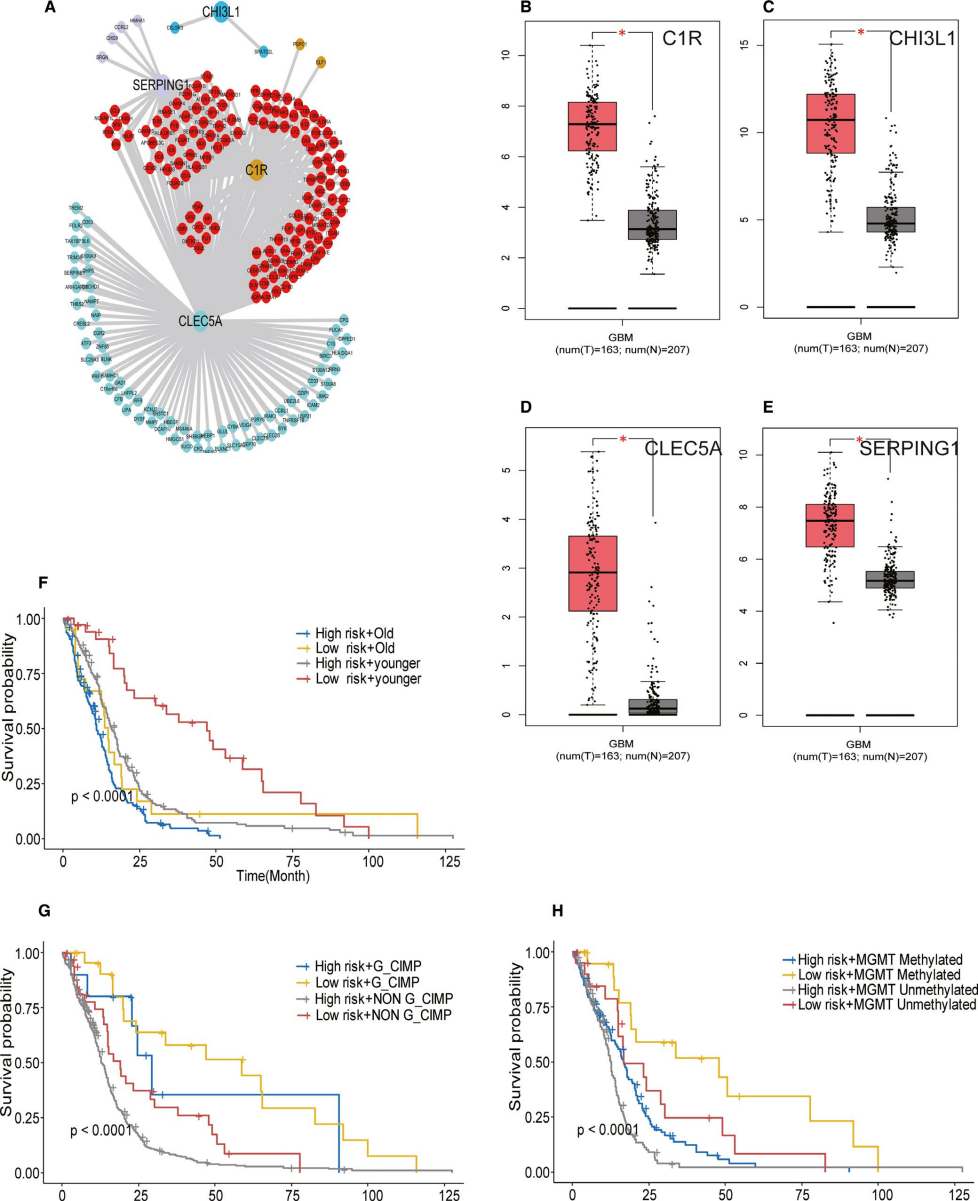
Co‐expression network of the TME‐related model and four‐gene model performance in different age groups: CIMP status and MGMT status in the TCGA cohorts. (A) The co‐expression network of the four‐gene model (C1R, CHI3L1, CLEC5A and SERPING1) is shown. Yellow, blue, green and light purple nodes represent the co‐expression genes related to C1R, CHI3L1, CLEC5A and SERPING1, respectively. Red nodes indicate the co‐expression genes, which interact at least two genes belonging to this model. (B‐E) Comparisons of the expression level of the selected four genes between GBM and non–GBM tissues in TCGA and GTEx based on GEPIA. The y‐axis represents the log2 (TPM + 1) for gene expression. The grey bar indicates the non–GBM tissues, and the red bar shows the GBM tissues. These figures were derived from GEPIA.TPM: transcripts per kilobase million. ∗*P* < .05. (F) Kaplan‐Meier survival curves for overall survival between younger group and old group in the TCGA cohort. (G) Kaplan‐Meier survival curves for overall survival between G‐CIMP group and non–G‐CIMP group in the TCGA cohort. (H) Kaplan‐Meier survival curves for the overall survival between MGMT‐methylated group and MGMT‐unmethylated group in the TCGA cohort

Through the robust likelihood‐based survival model and the LASSO method, four genes (*CLEC5A, SERPING1, CHI3L1* and *C1R*) were screened out to construct the TME‐related model. These four genes were also validated to be differentially expressed genes in the Gravendeel data set and another independent data set in the Chinese Glioma Genome Atlas (Table S1 and Figure S2B,C). A prognostic risk score for each patient was obtained by the regression coefficients and the transcriptome data of the genes. After calculating the risk scores, the TCGA GBM patients were separated into high‐ and low‐risk clusters according to the optimal cut‐off point of the risk scores. Figure 3A shows the distribution of the four‐gene signature risk scores. The survival status and survival time of the GBMs between the two risk groups are listed in Figure 3B. Notably, the expression of the four genes was remarkably higher in the high‐risk group than that in the low‐risk group (Figure 3C). Moreover, compared with the low‐risk group, the overall survival time of the high‐risk group was worse (*P < *0.0001) (Figure 3D). The area under a time‐dependent ROC curve (AUC) revealed that the 1‐, 3‐ and 5‐year overall survival of this model was 0.63, 0.70 and 0.71, respectively, in the training set (Figure 3E). The performance of this model in the Gravendeel validation data set was identical to that in the training set (Figure 3F‐I). The AUC of the signature for 1‐, 3‐ and 5‐year overall survival was 0.649, 0.840 and 0.821, respectively, in the validation set (Figure 3J). The co‐expression network was constructed after performing WGCNA of the training set using the R package of WGCNA, which elucidates the mechanism underlying these four genes.^18^ The correlations between gene modules and immune/stromal scores were measured by module eigengene (ME), which represents the expression level of the whole genes in corresponding modules. Twenty‐one gene modules were generated containing genes varying from 50 to 1085. Each module was tagged with a random colour for reference: black, blue, brown, cyan, dark red, green, green yellow, grey60, light cyan, light green, light yellow, magenta, midnight blue, pink, purple, red, royal blue, salmon, tan, turquoise and yellow. These modules contained 356, 691, 648, 82, 41, 474, 95, 68, 68, 63, 50, 187, 79, 208, 147, 380, 48, 83, 95, 1,085 and 535 genes, respectively. The ‘grey’ module was defined as a non–co‐expressed group according to the WGCNA developer's rationale (Figure S3A‐D). The results indicated that the yellow and brown modules contain the four genes, and genes belonging to these two modules were screened out. Based on genes contained in these two modules, the co‐expression network of the TME‐related model was built (Figure 4A). The number of genes co‐expressed with C1R, SERPING1, CLEC5A and CHI3L1 was 124, 48, 199 and 2, respectively. Containing 209 nodes, the co‐expression network of this model was highly connected by 373 edges (Figure 4A). GEPIA (http://gepia.cancer‐pku.cn/), a web server for analysing the RNA sequencing expression data of tumour and normal samples, revealed that the expression of these filtered four genes was expressed at a higher level in GBM than in non–GBM tissues (Figure 4B‐E).

### 3.4 Independent prognostic factor and hierarchic marker of TME‐related signatures

To identify whether the TME‐related signature is an independent predictive factor for GBM patients, the probable predictors, age group (young vs. old), CIMP status (non–G‐CIMP vs. G‐CIMP), MGMT status (unmethylated vs. methylated), subtype (neural + preneural vs. mesenchymal + classical) and risk level (low vs. high) were analysed by univariate and multivariate Cox proportional hazard regression. MGMT status (HR = 1.39, *P* = .017), CIMP status (HR = 3.04, *P* = .003), age group (HR = 0.63, *P* = .001) and risk level (HR = 0.53, *P* = .004) were found to be independent predictors in the TCGA GBM data set. CIMP status (HR = 2.09, *P* = .01), age group (HR = 0.46, *P* = 5.16 × 10^−05^) and risk level (HR = 0.64, *P* = .05) were independent predictors in the Gravendeel data set (Table 2). Our signature remained a remarkable prognostic biomarker after being adjusted by other predictive factors. When considering the combined effects of risk level and other clinicopathological factors (age, CIMP status, MGMT status), the younger patients in the low‐risk group exhibited the longest median survival, whereas the older patients in the high‐risk group showed the shortest median survival time (Figure 4F). Likewise, the high‐risk group patients harbouring non–G‐CIMP had the shortest median survival. In contrast, the G‐CIMP patients belonging to the low‐risk group showed the longest median survival time (Figure 4G). This outcome was verified in the validation data set (Figure S3E,F). Our model also stratified both MGMT‐methylated and MGMT‐unmethylated patients’ median survival remarkably well (Figure 4H). Therefore, these results suggest that the TME‐related prognostic model constructed is an independent prognostic biomarker and valuable marker for stratification in GBM.

**Table 2:** Univariate and multivariate cox proportional hazards analysis of clinical parameters and risk score level of GBM patients in the TCGA training set and Gravendeel validation set

Variables		Training set				Validation set			
		Univariate		Multivariate		Univariate		Multivariate	
		HR (95% CI)	*P*‐value	HR (95% CI)	*P*‐value	HR (95% CI)	*P*‐value	HR (95% CI)	*P*‐value
Age group	Younger vs old	0.54 (0.43‐0.67)	**2.04e‐08**	0.63 (0.48‐0.84)	**0.001**	0.37 (0.26‐0.52)	**2.63e‐08**	0.46 (0.32‐0.67)	**5.16e‐05**
CIMP status	Non‐ vs G‐CIMP	3.13 (2.01‐ 4.87)	**3.99e‐07**	3.04 (1.45‐6.35)	**0.003**	2.82 (1.71‐4.63)	**4.39e‐05**	2.09 (1.14‐3.83)	**0.01**
MGMT status	Un vs methylated	1.52 (1.16‐ 1.99)	**0.002**	1.39 (1.05‐1.83)	**0.017**	/	/	/	/
Subtype	NE + PN vs CL + ME	0.83 (0.66‐ 1.03)	0.092	1.16 (0.87‐1.55)	0.29	0.54 (0.38‐0.78)	**0.001**	1.15 (0.74‐1.78)	0.53
Risk level	Low vs High	0.45 (0.32‐ 0.62)	**2.29e‐06**	0.53 (0.34‐0.81)	**0.004**	0.44 (0.30‐0.65)	**3.73e‐05**	0.64 (0.41‐1.00)	**0.05**

### 3.5 The TME‐related prognostic model correlates with immune cell infiltration in GBM

**Figure 5:**
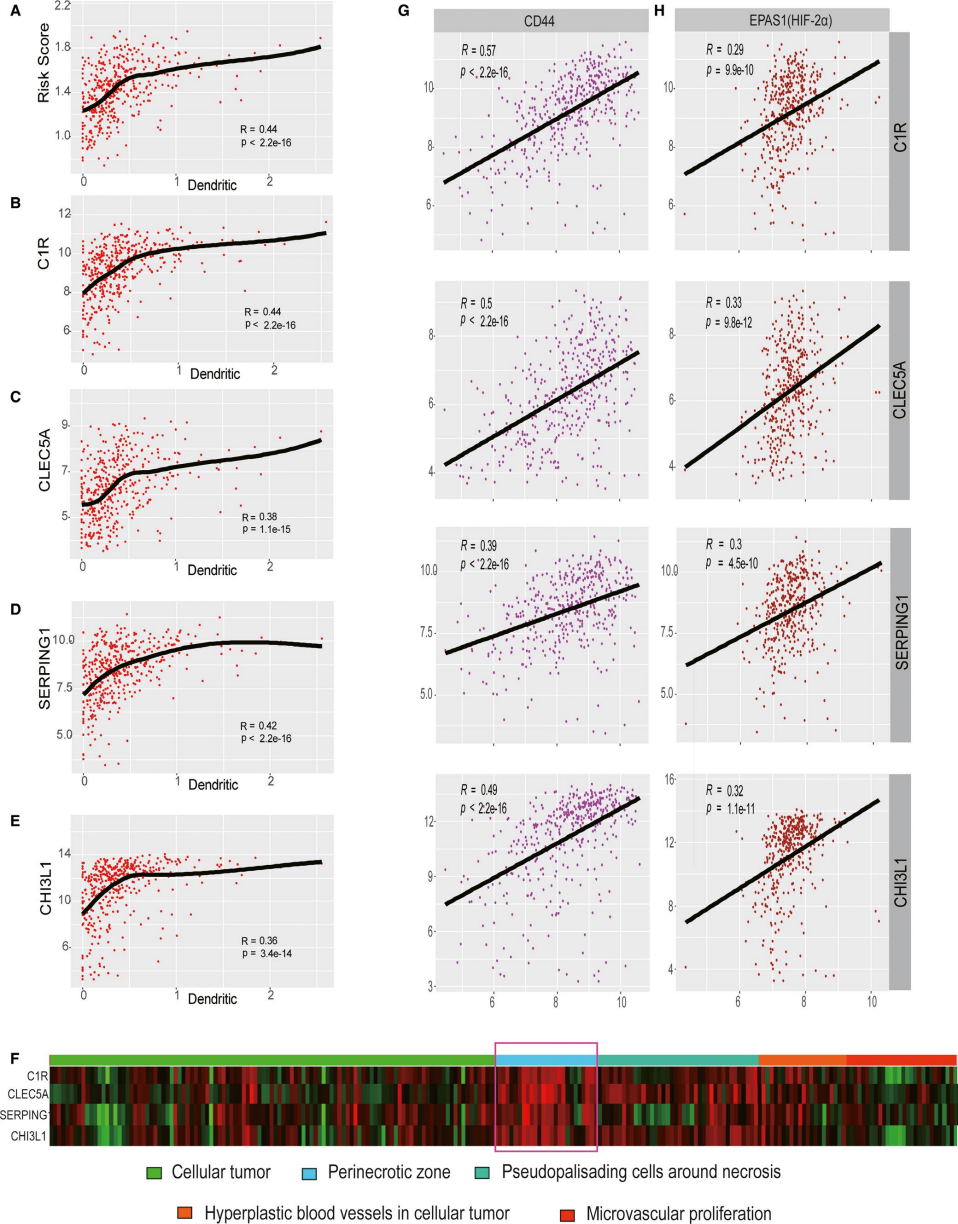
The correlation between model genes and risk score and dendritic cells and the TME‐related model might play an important role in the hypoxic phenotype of the perinecrotic GBM. The levels of risk score(A), C1R(B), CLEC5A(C), SERPING1(D) and CHI3L1(E) were significantly correlated with the infiltration levels of dendritic cells. (F) IVY GAP (http://glioblastoma.alleninstitute.org/static/home) analysis indicated that the expression of the TME‐related model was highly related to the perinecrotic zone in glioblastoma anatomic structures. (G) The expression level of this model was tightly correlated with the level of CD44. (H) The expression level of this model showed moderate correlation with the level of HIF‐2α

After constructing the TME‐related prognostic model using the training set and verifying its efficiency using the validation set, the correlation between this model and the infiltration of immune cells for GBM was calculated (Figure 5 and Figure S4). Scatter plots were generated using Pearson's correlation analysis, and statistical significance was determined. The risk score and the four identified genes in the model exhibited moderate correlation with the infiltration of dendritic cells (DCs) (*r* > 0.35, *P < *3.4 × 10^−14^) (Figure 5A‐E). CLEC5A presented a weak correlation with the infiltration of neutrophils (*r* = 0.19, *P* = 9.3 × 10^−05^), macrophages (*r* = 0.13, *P* = .0086) and CD4 + T cells (*r* = 0.15, *P* = .0019) (Figure S4A). SERPING1 exhibited a weak relationship with the infiltration of B cells (*r* = 0.14, *P* = .0047), CD4 + T cells (*r* = 0.14, *P* = .0034), CD8 + T cells (*r* = 0.14, *P* = .0039), neutrophils (*r* = 0.16, *P* = .0013) and macrophages (*r* = 0.16, *P* = .00074) (Figure S4B). CHI3L1 showed a weak correlation with the infiltration of CD4 + T cells (*r* = 0.16, *P* = .00099), neutrophils (*r* = 0.14, *P* = .0055) and macrophages (*r* = 0.13, *P* = .007) (Figure S4C). C1R displayed a weak correlation with the infiltration of B cells (*r* = 0.12, *P* = .013), CD4 + T cells (*r* = 0.18, *P* = .00033), CD8 + T cells (*r* = 0.17, *P* = 6 × 10^−04^), neutrophils (*r* = 0.17, *P* = .00059) and macrophages (*r* = 0.17, *P* = .00049) (Figure S4D). Risk scores presented a significant correlation with the infiltration of CD4 + T cells (*r* = 0.17, *P* = 5 × 10^−04^), CD8 + T cells (*r* = 0.097, *P* = .049), neutrophils (*r* = 0.2, *P* = 3.4 × 10^−05^) and macrophages (*r* = 0.15, *P* = .0016) (Figure S4E). This outcome reveals that our model system accounts for the immune cell infiltration in the GBM TME, especially the DCs.

### 3.6 The TME‐related genes might contribute to the hypoxic phenotype of perinecrotic GBM

To study the expression of the TME‐related genes in the GBM tissue, we explored the RNA‐seq data set of the Ivy Glioblastoma Atlas Project (http://glioblastoma.alleninstitute.org/static/home). This web server collected GBM samples laser‐dissected from various sites including cellular tumour, perinecrotic zone, pseudopalisading cells around necrosis, hyperplastic blood vessels in cellular tumours and proliferating microvasculature samples.^22^ Results showed that the GBM perinecrotic zone highly expressed TME‐related genes (Figure 5F). To further confirm this, we investigated the correlation between the model and perinecrosis‐related genes, such as CD44, which are activated under hypoxia and interact with HIF‐2α to modulate the hypoxic phenotype of perinecrotic and perivascular glioma cells.^23^ Scatter plots show that the expression of the four genes belonging to the TME‐related model exhibited a strong correlation with CD44 and HIF‐2α (known as EPAS1) (Figure 5G‐H). Additionally, the outcome of single‐cell clustering showed that these four genes were highly expressed either in three of the largest immune cell clusters defined by the pan‐immune marker *PTPCR* (*CD45*) or neoplastic cells that were collected from the tumour core, which is the location at which necrosis occurred (Figure S5A‐C). Immunohistochemistry results showed that C1R and CHI3L1 were highly expressed in the GBM perinecrotic zone compared with the non‐necrotic zone and peritumour tissue (Figure S6A,B). These indicate that the TME‐related genes might contribute to the hypoxic phenotype of perinecrotic GBM.

## 4 DISCUSSION

GBM occurs in the central nervous system, and the current treatment is ineffective. The TME plays a significant role in tumour malignancy and dictates therapeutic responses, including immunotherapy. Thus, a deeper knowledge of the TME in GBM can contribute to uncovering novel prognostic markers to administer precision immunotherapy. Current studies have been unable to successfully classify and analyse the components of tumour cells and the TME.^24^, ^25^, ^26^, ^27^, ^28^ Thus, we explored the TME components of GBM by comparing the gene expression data of 416 GBM patients with high and low stromal/immune scores and screening out 152 DEGRTME. Functional enrichment analyses were performed on the 152 genes; subsequently, a four‐gene TME‐related predictive model was constructed using the robust likelihood‐based survival model and the LASSO method. This model was verified to be an independent predictive biomarker after adjustment by important clinicopathological factors. Our study stands out from the previous ones, which filtered out hundreds of TME‐related genes, in that it provides a more precise targeted (*C1R, CLEC5A, SERPING1 and CHI3L1*) immunotherapy. In fact, some of them have been demonstrated to be oncogenes, and their increased expression has been found to be associated with poor survival in glioma patients. For example, CLEC5A is a member of the C‐type lectin/C‐type lectin‐like domain (CTL/CTLD) superfamily and is reported to be an M2 biomarker, associated with immunosuppression. CLEC5A overexpression decreases survival time in glioma patients.^29^ CHI3L1 encodes a glycoprotein member of the glycosyl hydrolase 18 family that is an immunomodulatory molecule, which may inhibit the PI3K/AKT pathway in GBM. High serum levels of CHI3L1 (also known as YKL‐40) are related to worse outcome.^30^ Our findings can provide novel molecular insights into the TME of GBM with these predictive genes acting as markers and/or therapeutic targets for the diagnosis and prediction of treatment outcome of GBM.

Recently, several genetic biomarkers, such as O(6)‐methylguanine‐DNA methyltransferase (MGMT) methylation, epidermal growth factor receptor variant III (EGFRvIII), vascular endothelial growth factor (VEGF) and isocitrate dehydrogenase (IDH), have been well‐established in GBM.^31^ However, these molecular markers still cannot completely explain the prognosis and treatment response (including immunotherapy) of GBM. Ho‐Keung Ng found that not all IDH‐mutant GBM subtypes have good prognosis, and it is a heterogeneous group that should be further stratified.^32^ It has been revealed that IDH‐mutant gliomas manifest the cytosine‐phosphate‐guanine (CpG) island methylator phenotype (G‐CIMP); therefore, we explored the classification efficiency of our model in G‐CIMP subsets of glioblastoma. The results showed that this risk model could efficiently stratify the G‐CIMP glioblastoma patients, and this stratification might have vital implications for bedside management.

An increasing number of investigations have suggested that the therapeutic effect of immunotherapies is correlated with immunogenic TME; DCs, which function as potential antigen‐presenting cells, have a central role in regulating immunity.^33^ In this study, a risk model was constructed based on differentially expressed genes related to TME. Moreover, the model itself and the genes belonging to the model exhibit moderate correlation with the infiltration of DCs, indicating that these genes could act as potential therapeutic candidates targeting DCs for immunotherapy of GBM and deserve to be studied further. The novelty of our study lies in using innovative methods to show that these genes might play a crucial role in the hypoxic phenotype of perinecrotic GBM. Perinecrosis refers to the hyperproliferative areas of GBM and comprises GBM stem cells (GSCs)/progenitors; it is also called as the perinecrotic niches. Studies have unearthed that GSCs can be induced by hypoxia and hypoxia‐inducible factor 2 (HIF‐2).^34^ CD44, activated under hypoxia, interacts with HIF‐2α to regulate the hypoxic phenotype of perinecrotic and perivascular glioma cells. Using the online tool, our study revealed that the four genes within the risk model were highly expressed in the perinecrotic zone of GBM. Furthermore, bioinformatic analysis indicated that the four genes presented strong and moderate correlation with the expression of CD44 and HIF‐2A, respectively. The findings of our study provide a solid foundation for subsequent in‐depth research in the development of prognostic biomarkers in GBM.

## 5 CONCLUSION

In conclusion, we constructed a four‐gene TME‐related predictive signature as an independent prognostic biomarker and compared it with other probable clinical features. The risk model showed good efficiency for GBM prognosis, verified by time‐dependent ROC curve analysis and survival analysis, and it was also a useful hierarchical marker for the G‐CIMP subtype of GBM. The four genes in our model might be important for the hypoxic phenotype of perinecrotic GBM and are remarkably correlated with infiltration of immune cells in the TME of GBM, indicating their potential as candidate targets for immunotherapy. However, the detailed molecular mechanisms underlying the functions of the four genes, which significantly influence the survival of GBM patients, have to be studied further.

## CONFLICT OF INTEREST

The authors declare that they have no competing interests.

## AUTHOR CONTRIBUTIONS

**Kai kai Xiao:** Data curation (equal); Methodology (lead); Software (lead); Visualization (lead); Writing‐original draft (lead). **Jun Tan:** Software (supporting); Visualization (equal); Writing‐review & editing (supporting). **Jian Yuan:** Visualization (equal); Writing‐review & editing (supporting). **Gang Peng:** Funding acquisition (equal); Visualization (supporting); Writing‐review & editing (supporting). **Wenyong Long:** Funding acquisition (equal); Writing‐review & editing (supporting). **Jun Su:** Visualization (equal); Writing‐review & editing (supporting). **Yao Xiao:** Visualization (equal); Writing‐original draft (supporting). **Qun Xiao:** Methodology (supporting); Visualization (supporting). **Changwu Wu:** Methodology (supporting); Visualization (supporting). **Chaoying Qin:** Visualization (supporting); Writing‐review & editing (supporting). **Lili Hu:** Software (supporting); Visualization (supporting). **Kaili Liu:** Software (supporting); Visualization (supporting). **Shunlian Liu:** Software (supporting); Visualization (supporting). **Hao Zhou:** Software (supporting); Visualization (supporting). **Yichong Ning:** Software (supporting); Visualization (supporting). **Xiaofeng Ding:** Project administration (supporting); Visualization (equal); Writing‐review & editing (equal). **Qing Liu:** Data curation (lead); Funding acquisition (lead); Project administration (lead); Visualization (equal).

## Supporting information

Fig S1‐S6

Table S1

## Data Availability

The data sets of this study were generated from the GlioVis (http://gliovis.bioinfo.cnio.es/), ESTIMATE (https://bioinformatics.mdanderson.org/estimate/), CGGA(http://www.cgga.org.cn/) and TIMER (https://cistrome.shinyapps.io/timer/). The single‐cell RNA‐seq data set was downloaded from http://www.gbmseq.org/. The code supporting the current study is available from the corresponding author on request.
