# Supplementary material for: Prognostic value and immune cell infiltration of hypoxic phenotype‐related gene signatures in glioblastoma microenvironment
Source: J Cell Mol Med. 2020 Oct 3;24(22):13235–47. doi: 10.1111/jcmm.15939 (PMC7701576; doi:10.1111/jcmm.15939)
Supplement: Supplementary file 1 — Fig S1‐S6 [file JCMM-24-13235-s001.docx]

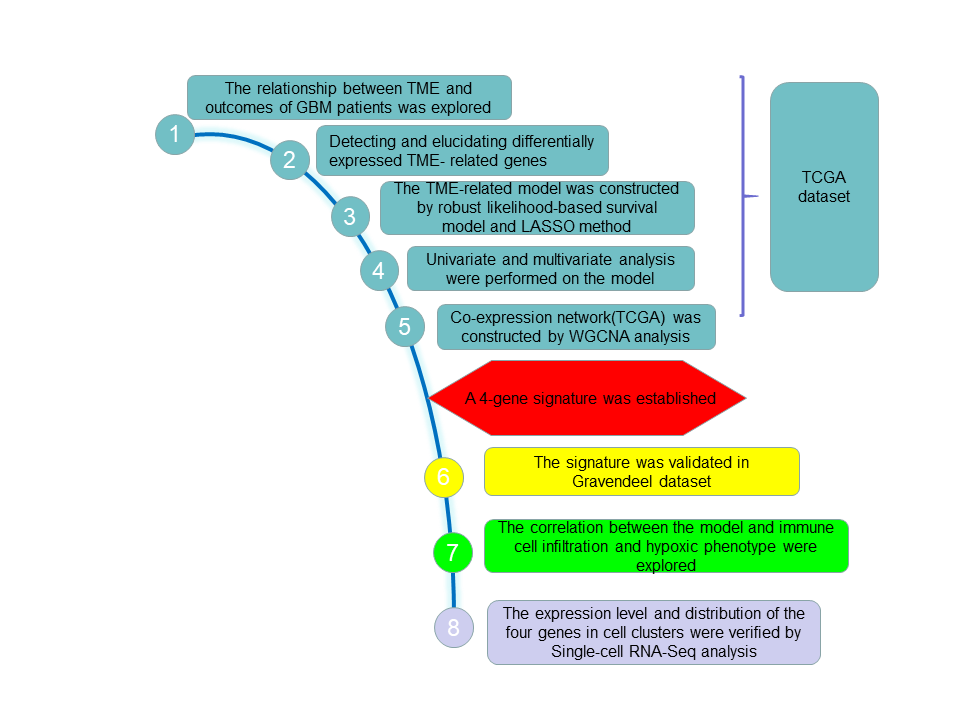


Supplemental Figure 1

Study outline. The outline indicates the exploration process.


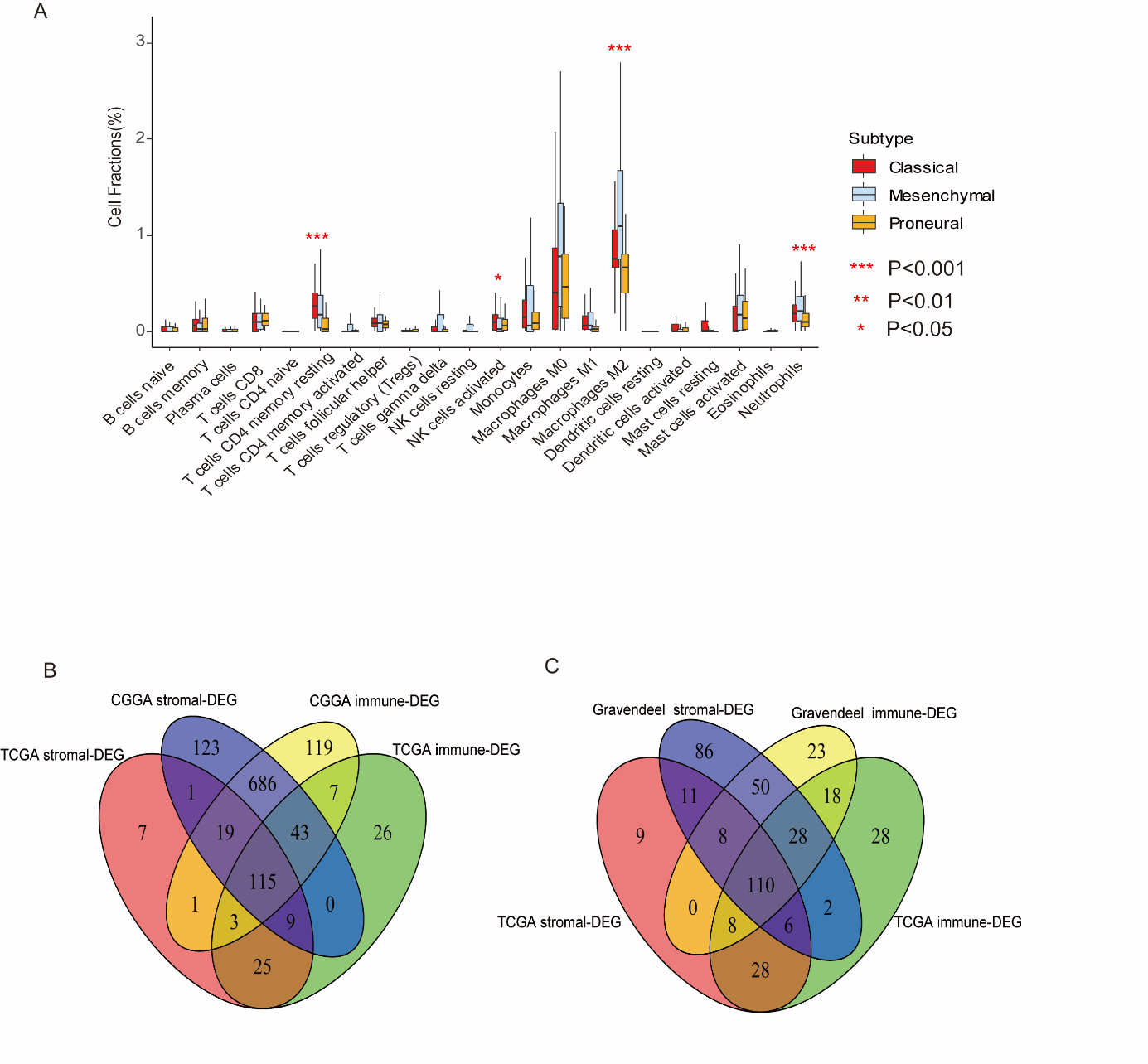


Supplemental Figure 2

Transcriptional subtypes differentially activate the immune microenvironment and the overlap of differentially expressed TME-related genes in different datasets. (A) A comparison of the immune cell fractions among subtypes. The Venn diagrams show the overlap genes of the TCGA and CGGA dataset (B) and the TCGA and Gravendeel dataset (C).


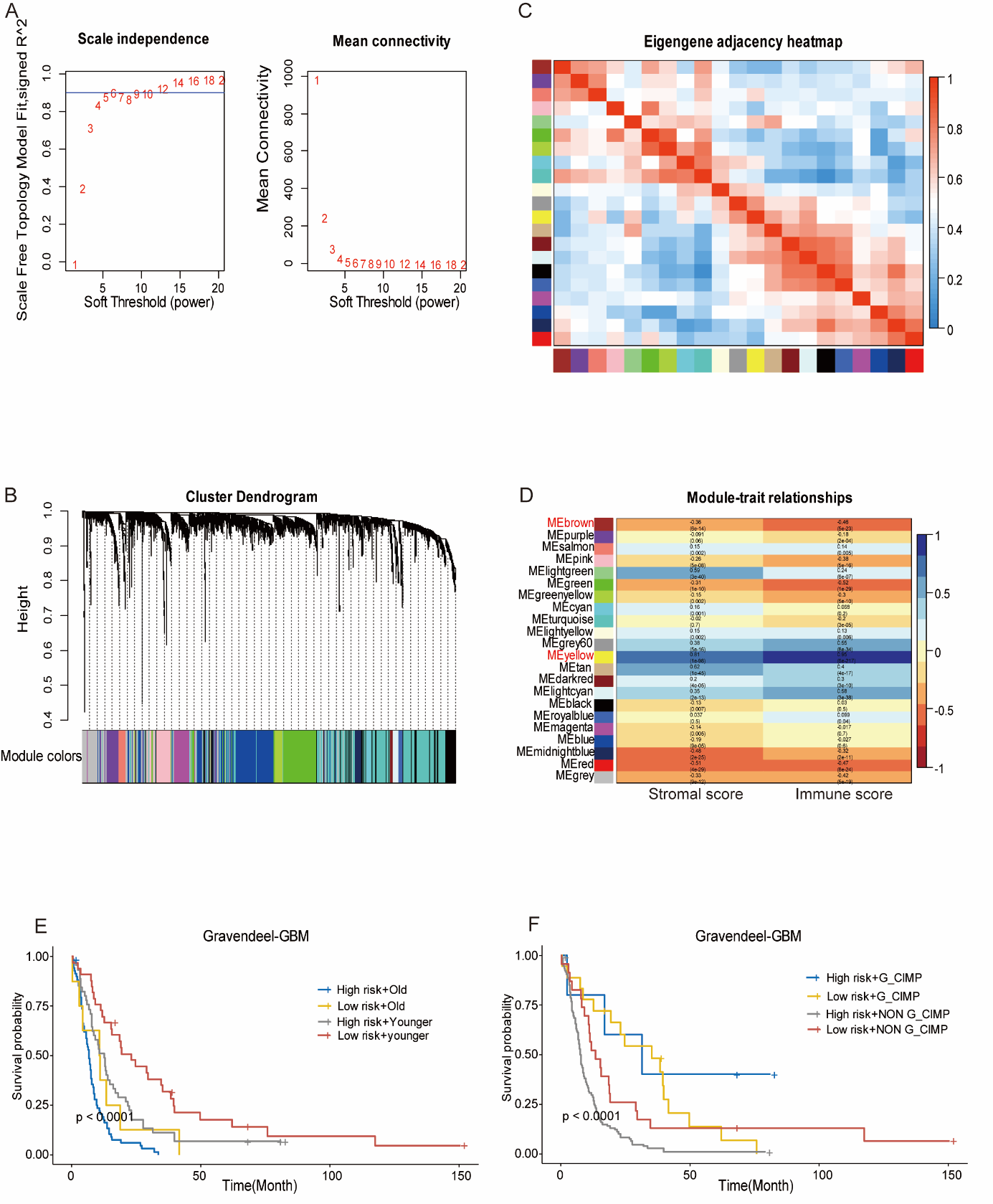


Supplemental Figure 3

Weighted gene correlation network analysis of TCGA GBM and the four-gene model performance for different age groups. CIMP status in the Gravendeel cohorts. (A) Identification of the soft threshold according to the standard of the scale-free network. (B) Identification of a co-expression module in GBM. The branches of the cluster dendrogram correspond to the 21 different gene modules. Each piece of the leaves on the cluster dendrogram corresponds to a gene. (C) The eigengene adjacency heatmap of 21 co-expression models. (D) Correlation between the gene module and immune/stromal score. The correlation coefficient in each cell represented the correlation between the gene module and immune/stromal score, which decreased in size from blue to red. The corresponding P value is annotated. (E) Kaplan–Meier survival curves for overall survival between the younger and old groups in the Gravendeel cohort. (F) Kaplan–Meier survival curves for overall survival between the G-CIMP group and NON G-CIMP group in the Gravendeel cohort.


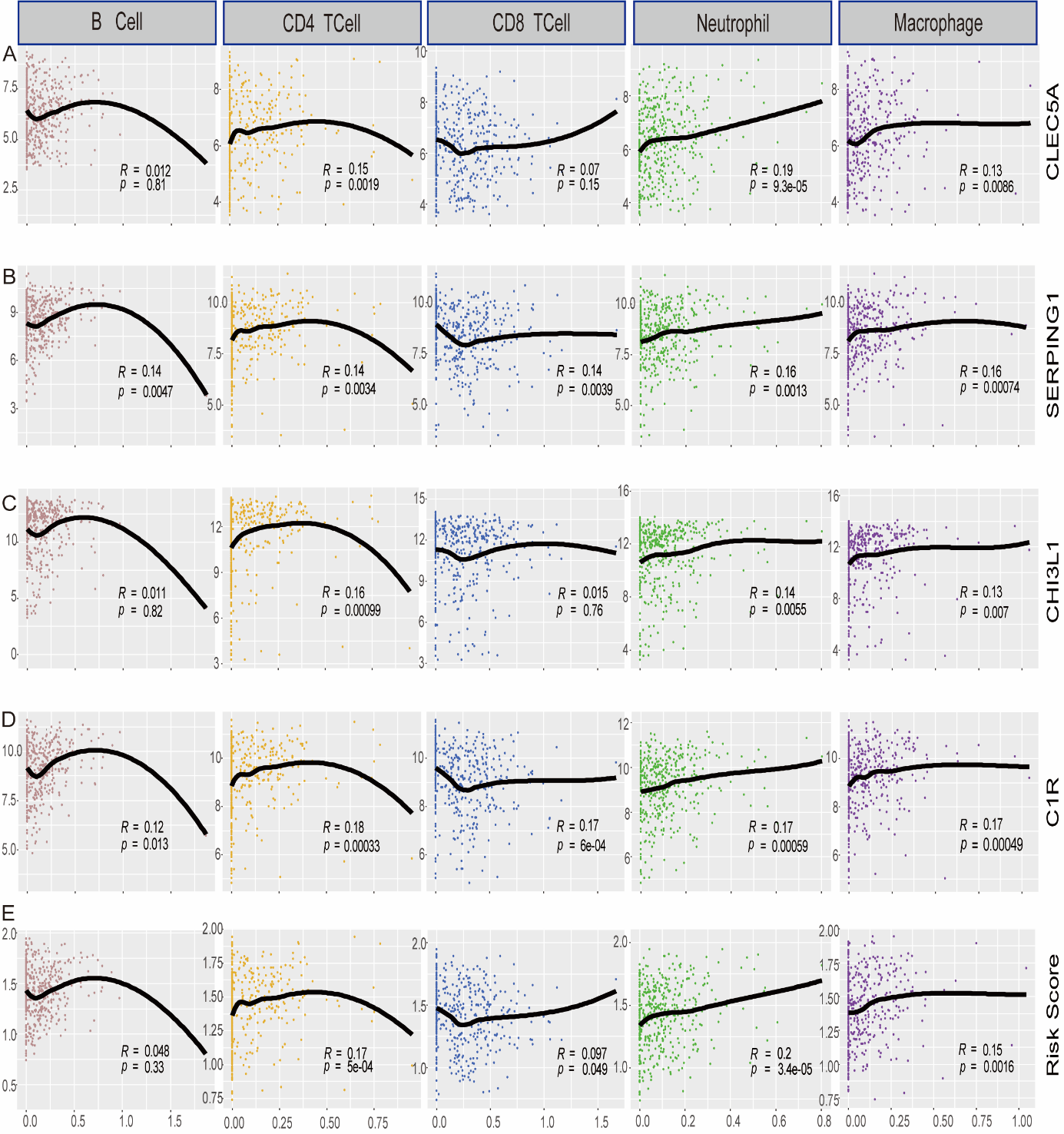


Supplemental Figure 4

Correlation between model genes(A-D) as well as the risk score(E) and immune cells except dendritic cells.


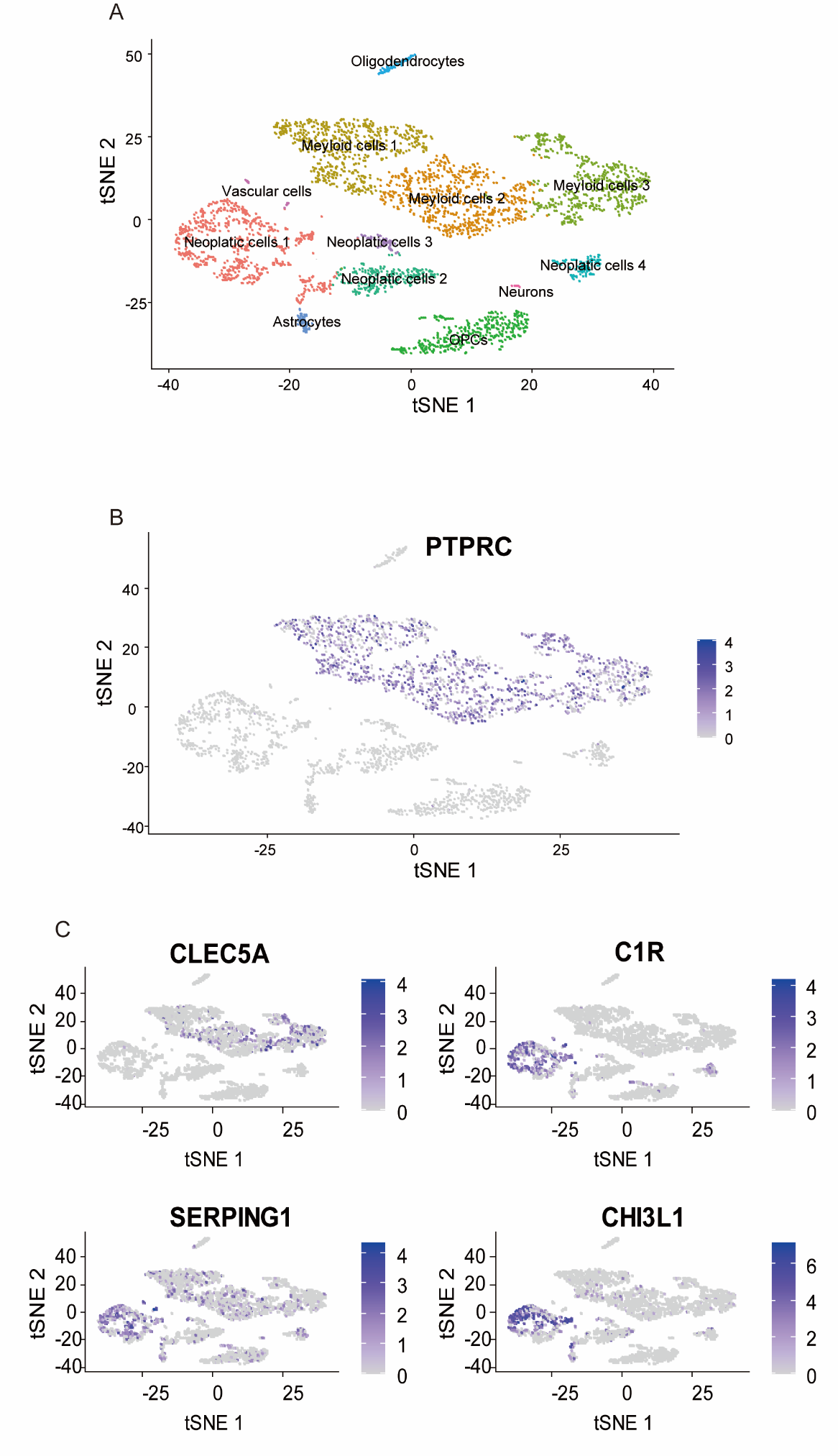


Supplemental Figure 5

Classification of single cells and annotation of the four genes in cell clusters. (A)2D-tSNE representation of all single cells included in the study (n = 3,589). Cell clusters are differentially colored and identified as distinct cell classes. (B) Expression of pan-immune marker PTPRC (CD45) overlaid on the 2D-tSNE space. (C) Expression of the four genes within the model overlaid on the 2D-tSNE space.


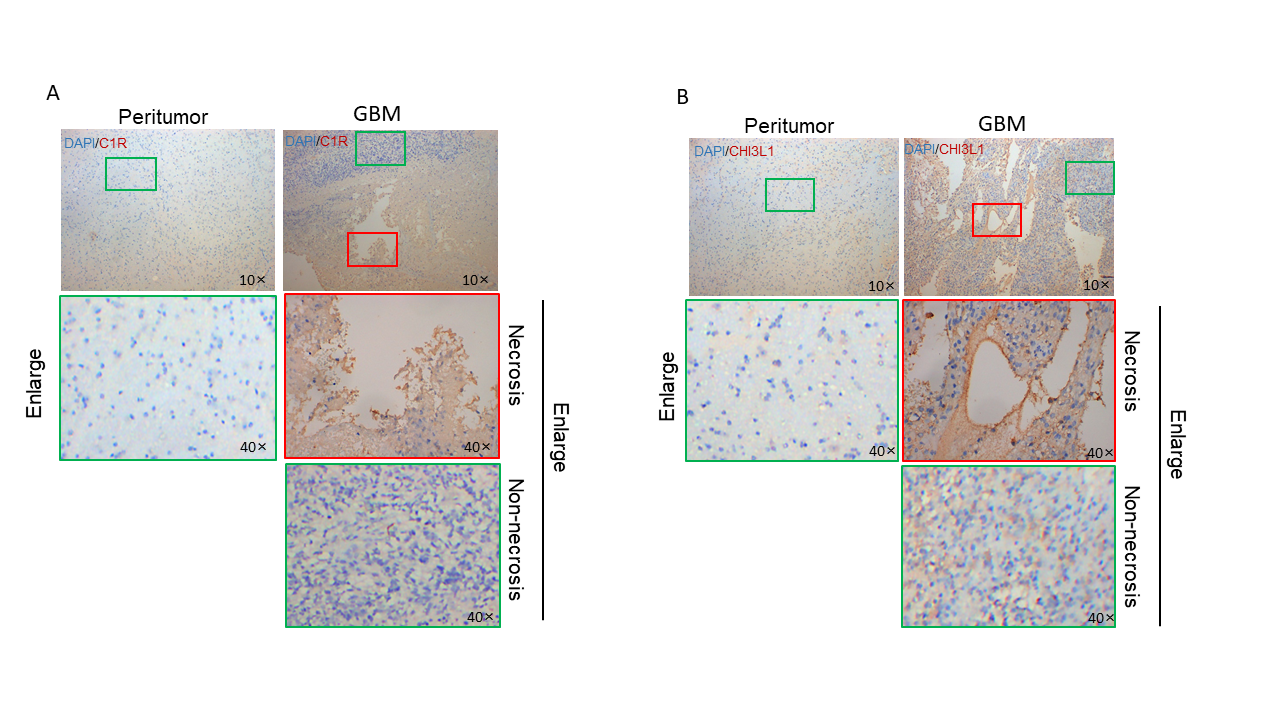


Supplemental Figure 6

C1R(A) and CHI3L1(B) protein expression in the peritumor tissue and GBM necrosis/non-necrosis sites. Immunohistochemistry images of proteins (C1R and CHI3L1) detected in GMB and the peritumor sample which showed almost negative staining in the peritumor tissue but rather high expression in the GBM tissue, especially in the perinecrotic zone.
